# Overexpressed WDR3 induces the activation of Hippo pathway by interacting with GATA4 in pancreatic cancer

**DOI:** 10.1186/s13046-021-01879-w

**Published:** 2021-03-01

**Authors:** Wenjie Su, Shikai Zhu, Kai Chen, Hongji Yang, Mingwu Tian, Qiang Fu, Ganggang Shi, Shijian Feng, Dianyun Ren, Xin Jin, Chong Yang

**Affiliations:** 1 Department of Anesthesiology, Sichuan Provincial People’s Hospital, University of Electronic Science and Technology of China, Chengdu, 611731 Sichuan China; 2 Clinical Immunology Translational Medicine Key Laboratory of Sichuan Province & Organ Transplantation Center, Sichuan Provincial People’s Hospital, University of Electronic Science and Technology of China, Chengdu, 611731 Sichuan China; 3 Chinese Academy of Sciences Sichuan Translational Medicine Research Hospital, Chengdu, 610072 Sichuan China; 4 Transplant Center, Massachusetts General Hospital, Harvard Medical School, Boston, MA 02148 USA; 5 Jack Bell Research Centre, University of British Columbia, Vancouver, BC V6H3Z6 Canada; 6 Department of Pancreatic Surgery, Union Hospital, Tongji Medical College, Huazhong University of Science and Technology, Wuhan, 430022 Hubei China

**Keywords:** Pancreatic Cancer, WDR3, GATA4, YAP1, Hippo signaling pathway

## Abstract

**Background:**

WD repeat domain 3 (WDR3) is involved in a variety of cellular processes including gene regulation, cell cycle progression, signal transduction and apoptosis. However, the biological role of WDR3 in pancreatic cancer and the associated mechanism remains unclear. We seek to explore the immune-independent functions and relevant mechanism for WDR3 in pancreatic cancer.

**Methods:**

The GEPIA web tool was searched, and IHC assays were conducted to determine the mRNA and protein expression levels of WDR3 in pancreatic cancer patients. MTS, colony formation, and transwell assays were conducted to determine the biological role of WDR3 in human cancer. Western blot analysis, RT-qPCR, and immunohistochemistry were used to detect the expression of specific genes. An immunoprecipitation assay was used to explore protein-protein interactions.

**Results:**

Our study proved that overexpressed WDR3 was correlated with poor survival in pancreatic cancer and that WDR3 silencing significantly inhibited the proliferation, invasion, and tumor growth of pancreatic cancer. Furthermore, WDR3 activated the Hippo signaling pathway by inducing yes association protein 1 (YAP1) expression, and the combination of WDR3 silencing and administration of the YAP1 inhibitor TED-347 had a synergistic inhibitory effect on the progression of pancreatic cancer. Finally, the upregulation of YAP1 expression induced by WDR3 was dependent on an interaction with GATA binding protein 4 (GATA4), the transcription factor of YAP1, which interaction induced the nuclear translocation of GATA4 in pancreatic cancer cells.

**Conclusions:**

We identified a novel mechanism by which WDR3 plays a critical role in promoting pancreatic cancer progression by activating the Hippo signaling pathway through the interaction with GATA4. Therefore, WDR3 is potentially a therapeutic target for pancreatic cancer treatment.

**Supplementary Information:**

The online version contains supplementary material available at 10.1186/s13046-021-01879-w.

## Background

Pancreatic ductal adenocarcinoma (PDAC) is a highly malignant disease with a 5-year survival rate of only 10% for all stages combined [1]. According to GLOBOCAN 2020 estimates, PDAC is the seventh leading cause of cancer-related mortality worldwide and accounting for about 495,773 new cases and 466,003 deaths [2]. Surgical resection remains the only treatment that offers a curative potential, whereas most cases lost the chance because of those presenting with advanced stages at the time of diagnosis [3]. Currently, molecular targeted therapy showed great potential for improving the survival rates of PDAC patients [4]. Then, it is significant for developing new therapeutic strategies and potential therapeutic targets for pancreatic cancer treatment.

WD repeat domain 3 (WDR3), also known as DIP2 or UTP12, belongs to the WD-repeat family and is a component of the 80 S complex of the small subunit processome, which is implicated in the 40 S ribosome synthesis pathway [5]. It has been reported that WDR3 is involved in a variety of cellular processes including genome stability, cell proliferation, signal transduction, and apoptosis [6–8]. McMahon et al proved that suppression of WDR3 reduced breast carcinoma cell proliferation and focus formation [7]. Also, Akdi et al indicated WDR3 gene expression is associated with thyroid cancer risk in special populations [9], and WDR3 can modulate genome stability in thyroid cancer patients [10]. These studies revealed WDR3 confer growth and proliferative advantages of some malignant cancer, whereas the biological role of WDR3 in pancreatic cancer and the relevant mechanism remain unclear.

Studies proved the Hippo signaling pathway plays a critical role in modulating cell proliferation and has been demonstrated to contribute to the progression of malignant cancers, including pancreatic cancer [11–14]. The core components of the Hippo signaling pathway, including yes association protein 1 (YAP1), promote the migration, invasion, and malignancy of cancer cells [15], and inhibiting YAP1 expression suppresses pancreatic cancer progression by disrupting tumor-stroma interaction [16, 17]. YAP1 usually enters the nucleus and interacts with other transcription factors, including TEA domain (TEAD) family members, to regulate downstream gene targets [18–20]. Study of connective tissue growth factor (CTGF) and cysteine rich angiogenic inducer 61 (CYR61), the major downstream gene targets regulated by YAP1, has provided new insights into the physiological/pathological functions of Hippo pathway effectors [12, 21]. CYR61 and CTGF had been reported to act as factors stimulating aggressiveness in a variety of cancers. CYR61 expression was exorbitantly higher in cancer cells and significantly triggered the aggressive phenotype in PADC [22]. CTGF was a fibrosis-related gene related to pancreatic cancer progression by protecting pancreatic cancer cells from hypoxia-mediated apoptosis, and tumor cell-derived CTGF was vital for pancreatic cancer growth [23]. Therefore, exploring methods to inhibit YAP1 expression is essential for improving pancreatic cancer therapy.

In our study, we proved that overexpressed WDR3 was correlated with poor survival in pancreatic cancer patients. Furthermore, WDR3 silencing could significantly decrease the proliferative and invasive abilities of pancreatic cancer cells by inducing YAP1 inhibition, which was found to rely on the interaction between WDR3 and GATA4. Taken together, our results emphasize the importance of WDR3 as a therapeutic target in pancreatic cancer.

## Materials and methods

### Cell culture

The PANC-1, MIA PaCa-2, and BxPC-3 cell lines were purchased from the Type Culture Collection Cell Bank of the Chinese Academy of Sciences (Shanghai, China). PANC-1 and MIA PaCa-2 cells were cultured in Dulbecco’s Modified Eagle’s Medium (DMEM) (#30030, Thermo Fisher Scientific) supplemented with 10% fetal bovine serum (FBS) (#10099141, Thermo Fisher Scientific) and 1% penicillin/streptomycin at 37 °C in a 5% CO_2_ incubator. BxPC-3 cells were cultured in RPMI-1640 medium (#88365, Thermo Fisher Scientific) supplemented with10% FBS and 1% penicillin/streptomycin at 37 °C in a 5% CO_2_ incubator.

### Antibodies and chemicals

An anti-WDR3 antibody (#ab176817, working dilution 1:1000) was purchased from Abcam. An anti-GAPDH antibody (#10494–1-AP, working dilution: 1:3000), anti-GATA4 antibody (#19530–1-AP, working dilution: 1:1000), and anti-YAP1 antibody (#13584–1-AP, working dilution: 1:1000) were acquired from Proteintech. TED-347 (HY-125269, working concentration: 10 μM) was procured from MedChemExpress (USA).

### Immunoprecipitation and western blot analysis

Whole cell lysates were obtained with RIPA lysis buffer (Cell Signaling Technology, Danvers, MA) containing 1% protease and phosphatase inhibitors (Sigma-Aldrich) on ice. The resulting cell lysates were centrifuged at 12,000 rpm for 15 min at 4 °C to remove undissolved impurities and collect the supernatants. The protein concentration was quantified using a BCA assay (#P0012S, Beyotime). Protein extracts (500 μg) were incubated with appropriate primary antibody beads overnight for an immunoprecipitation assay or directly evaluated for western blot analysis. The precipitated immune complexes were subjected to SDS-PAGE, transferred to 0.45-μm polyvinylidene difluoride (PVDF) membranes, and then immunoblotted with specific primary antibodies. The signal intensities of bands were quantified using ImageJ software.

### Liquid chromatography-tandem mass spectrometry/mass spectrometry (LC-MS/MS) analysis

To identify potential WDR3-binding proteins, 293 T cells transduced with pcDNA3-WDR3 were collected for assays. WDR3 was pulled down by IP using an anti-WDR3 antibody and protein A + G agarose (#P2012, Beyotime) at 4 °C. LC-MS/MS analysis was performed using a Thermo Ultimate3000 liquid phase combined with Q Exactive Plus high-resolution mass spectrometry at Shanghai Applied Protein Technology. The data were retrieved with maxquant (v1.6.6) software, and the database retrieval algorithm was Andromeda. The database used in the search was the human proteome reference database of UniProt. The results were screened with a 1% FDR at the protein and peptide levels.

### RNA-seq

A total amount of 1 μg of RNA per sample was used as the input material for RNA sample preparations. Sequencing libraries were generated using the NEBNext® UltraTM RNA Library Prep Kit for Illumina® (NEB, USA) following the manufacturer’s recommendations, and index codes were added to attribute sequences to each sample. Clustering of the index-coded samples was performed on the cBot Cluster Generation System using the TruSeq PE Cluster Kit v3-cBot-HS (Illumina) according to the manufacturer’s instructions. After cluster generation, the library preparations were sequenced on an Illumina Novaseq platform, and 150-bp paired-end reads were generated. FeatureCounts v1.5.0-p3 was used to count the read numbers mapped to each gene. Differential expression analysis of two conditions/groups (two biological replicates per condition) was performed using the DESeq2 R package (1.16.1). We used the cluster Profiler R package to test the statistical enrichment of differentially expressed genes in KEGG pathways.

### Quantitative RT-PCR assay

Total RNA was extracted with RNAiso Plus (#15596026, Invitrogen). The PrimeScript RT Reagent Kit (#RR047A, TAKARA, Japan) was used for reverse transcription. Real-time PCR (RT-PCR) was conducted with a TB Green™ Fast qPCR Mix kit (#RR430A, TAKARA, Japan). The 2^-ΔCt^ method was used to quantify fold changes with normalization to GAPDH. Detailed information on the primer sequences is shown in Table S1.

### RNA interference

Sh-Control and gene-specific shRNAs were procured from Sigma-Aldrich, and si-Control and gene-specific siRNAs were provided by RiboBio. Pancreatic cancer cells were transfected with siRNA using Lipofectamine 2000 (#11668019, Thermo Fisher Scientific) in accordance with the manufacturer’s instructions for 24 h, and then the Lipofectamine 2000-containing medium was replaced with fresh DMEM containing 10% FBS. 293 T cells were transfected with shRNA plasmids and packaging plasmids (pVSV-G and pEXQV) in Lipofectamine 2000 according to the manufacturer’s instructions for 24 h, and the Lipofectamine 2000-containing medium was replaced with fresh DMEM containing 10% FBS and 1 mM sodium pyruvate. At 48 h post transfection, the virus culture medium was collected and added to pancreatic cancer cells for 24 h of culture, after which the infected cells were selected with 1 μg/ml puromycin. The shRNA and siRNA sequences are shown in Table S2.

### Chromatin immunoprecipitation (ChIP) and ChIP-qPCR

ChIP was performed with the Chromatin Extraction Kit (#ab117152, Abcam) and ChIP Kit Magnetic-One Step (#ab156907, Abcam) according to the manufacturer’s instructions. Purified DNA was analyzed using RT-PCR with a TB Green™ Fast qPCR Mix kit (#RR430A, TAKARA, Japan) following the manufacturer’s protocols. The ChIP-qPCR primers are shown in Table S3.

#### Nuclear and cytoplasmic extracts preparation

Cells were collected and the cell pellet was resuspended in 1 mL of Buffer A (10 mM HEPES-KOH, pH 7.9, 1.5 mM MgCl2, 10 mM KCl, 0.1% NP-40) to lyse the cells on ice for 10 min. Samples were spined down at 6500 rpm 4 °C for 3 min to pellet the nuclei. Nuclei pellet was washed with Buffer A and spined down at 3500 rpm for 5 min at 4 °C. The cell pellet was lysed by IP buffer (50 mM Tris-HCl, pH 7.4, 150 mM NaCl, 1% Triton X-100, 1% sodium deoxycholate, and 1% protease inhibitor cocktails) on ice for more than 30 min. Protein concentration was determined by BCA protein quantification assay.

### Colony formation assay

For colony formation assays, 500 pancreatic cancer cells transfected with sh-Control or sh-WDR3s were seeded in a six-well plate and cultured for approximately 10–12 days. Then, the colonies were fixed in methanol for 30 mins and stained with a 4 g/l crystal violet solution for 30 mins. The colonies were photographed, and the number of colonies was counted. All assays were performed in triplicate.

### MTS assay

For MTS assays, transfected pancreatic cancer cells were seeded in 96-well plates with 2500 cells per well. After 72 h of culture, [3-(4,5-dimethylthiazol-2-yl)-5-(3-carboxymethoxyphenyl)-2-(4-sulfophenyl)-2H-tetrazolium] (MTS reagent) (Abcam, #ab197010, USA) was added to each well for three hours of culture according to the manufacturer’s instructions. The absorbance in each well was measured with a microplate reader at 490 nm. Each experiment included five replicates and was performed in triplicate.

### Cell invasion assay

Cell invasion assays were performed using transwell chambers (8-μm pore size; Millipore) with a Matrigel (BD Biosciences, CA, USA) matrix. In brief, 600 μl of complete medium supplemented with 30% FBS was added to the bottom chamber, and 10^5^ transfected pancreatic cancer cells were suspended in 200 μL of serum-free medium and added to the upper chamber. After culturing for 12–24 h, the cells on the top surface of the membrane were mechanically removed using a cotton swab. The cells on the bottom surface of the membrane were fixed in methanol for 30 mins and stained with a 4 g/l crystal violet solution for 30 mins. The invaded cells were counted under a microscope, with five fields per well evaluated. Each experiment was performed in triplicate.

### Bioinformatic mining

Gene correlation analyses between the mRNA expression levels of WDR3 and YAP1 were carried out with the GEPIA database (http://gepia.cancer-pku.cn/) for all given sets of GTEx and TCGA expression data. The Eukaryotic Promoter Database (https://epd.epfl.ch//index.php) was used to determine the potential binding sites of GATA4 in the promoter of the YAP1 gene.

### PDAC xenografts in nude mice

Animal experiments were approved by the Ethical Committee on Animal Experiments of the Sichuan Provincial People’s Hospital in Chengdu, China. PANC-1 cells (3 × 10^6^) infected with sh-Control or sh-WDR3 #1 were subcutaneously injected into the left flank of BALB/c-nu mice (4–5 weeks old, male) purchased from Vital River. Tumor sizes were assessed with a digital Vernier caliper every three days. Tumors were harvested 3 weeks after injection, and tumor weights were measured.

### Orthotopic syngeneic model of pancreatic cancer to C57BL/6 mice

We used 8-week-old wild-type C57BL/6 mice in the experiments. For orthotopic implantation, mice were anesthetized with pentobarbital sodium, and hair was removed from their abdomens. We incised each mouse longitudinally along the abdomen to expose the pancreas, injected 20 μL of the cell suspension into the pancreas, and closed the incision with sutures. Each experimental group consisted of five mice. All mice were weighed and checked for signs of distress regularly. Abdominal palpation was used to monitor tumor size. Tumors were harvested 3 weeks after injection, and tumor weights were measured.

### Statistical analysis

All data are expressed as the mean ± standard deviation (SD) of three independent experiments. Comparisons between two groups were performed using Student’s t-test, and two-way ANOVA or one-way ANOVA together with the Bonferroni post hoc test was used for multigroup analysis. A *P* value less than 0.05 was considered significant. GraphPad Prism 6 software (GraphPad Software, Inc.) was used for statistical analysis.

## Results

### Overexpressed WDR3 was correlated with poor survival in pancreatic cancer patients

**Fig. 1:**
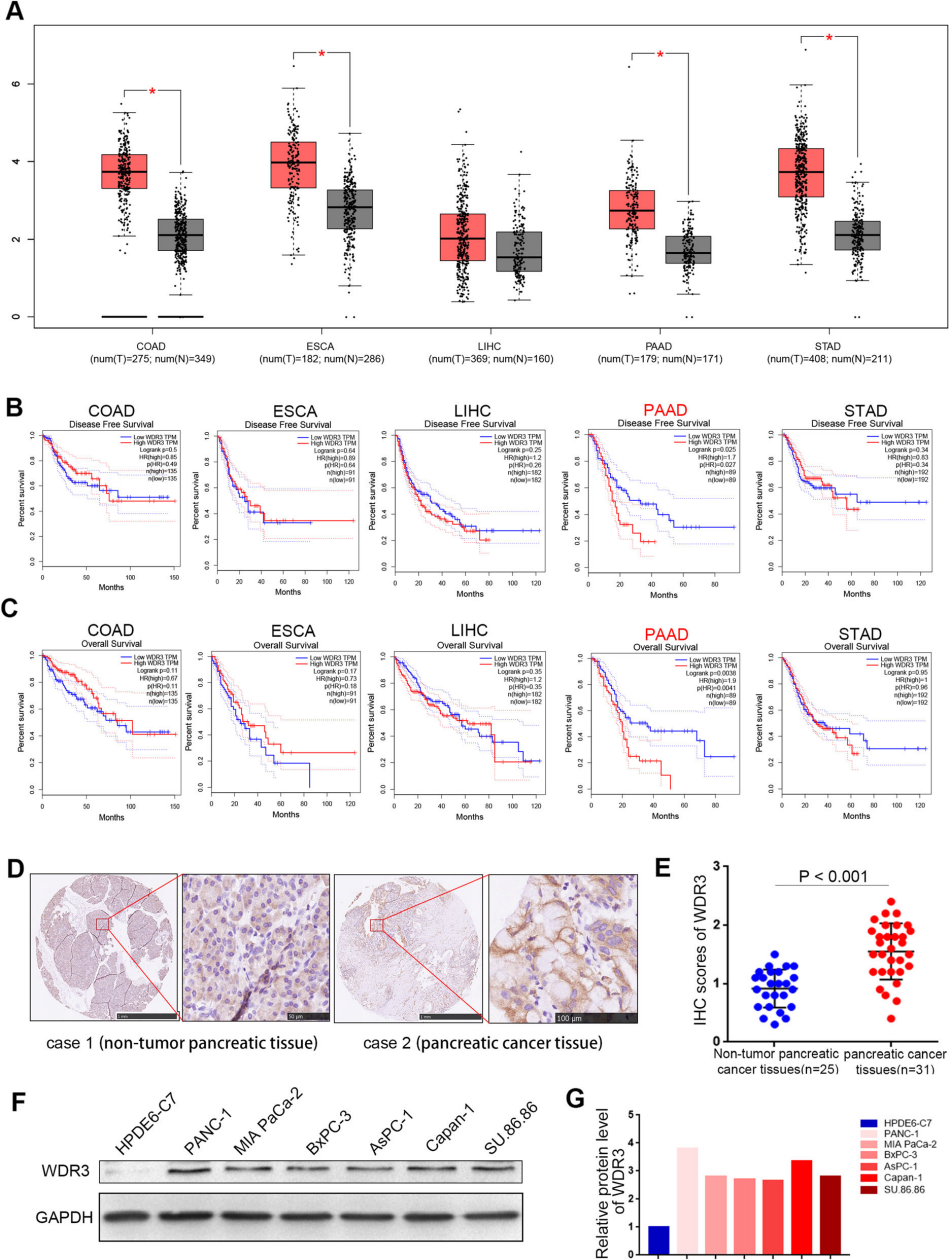
Overexpression of WDR3 is correlated with an unfavorable prognosis in pancreatic cancer. **a** The GEPIA database was searched for WDR3 mRNA expression in several malignant cancers. Colon adenocarcinoma (COAD), esophageal carcinoma (ESCA), liver hepatocellular carcinoma (LIHC), pancreatic adenocarcinoma (PAAD), and stomach adenocarcinoma (STAD). *, *P* < 0.05. **b-c** The GEPIA web tool was searched for disease-free survival (**b**) and overall survival (**c**) data for several malignant cancer patients with a high or low WDR3 expression level. *P* values are shown in the Fig. **d.** IHC images of WDR3 staining in TMA tissue sections are shown. The scale bars are shown in the Fig. **e.** Dot plots show the IHC scores of WDR3 expression for TMA tissue sections (normal pancreatic specimens: *n* = 25, PAAD TMA specimens: *n* = 31, *P* < 0.001). Statistical analyses were performed with D’Agostino & Pearson omnibus normality test. **f** and **g** Western blot analysis evaluated the expression of WDR3 in normal pancreatic ductal epithelial cells and pancreatic cancer cells (**f**). The protein expression levels of WDR3 were quantified with ImageJ software (**g**). GAPDH served as an internal reference

To determine the expression level of WDR3 in malignant cancers, the GEPIA database was searched, and the results showed that WDR3 was significantly overexpressed in several malignant cancers, including pancreatic cancer (Fig. 1a). Moreover, survival rate analysis results indicated that the elevated expression level of WDR3 was correlated with poor disease-free survival (DFS) and overall survival (OS) in pancreatic cancer, rather than other malignant cancers (Fig. 1b-c). Therefore, we speculated that WDR3 plays an important biological role in the progression of pancreatic cancer. Furthermore, IHC analysis verified the overexpression of WDR3 in pancreatic cancer patients (Fig. 1d-e). Finally, compared with normal pancreatic cells, pancreatic cancer cells expressed high levels of WDR3 (Fig. 1f-g). Therefore, we concluded that WDR3 was significantly overexpressed and positively correlated with poor survival in pancreatic cancer.

### Silenced WDR3 inhibited the proliferation and invasion of pancreatic cancer cells in vitro and in vivo

**Fig. 2:**
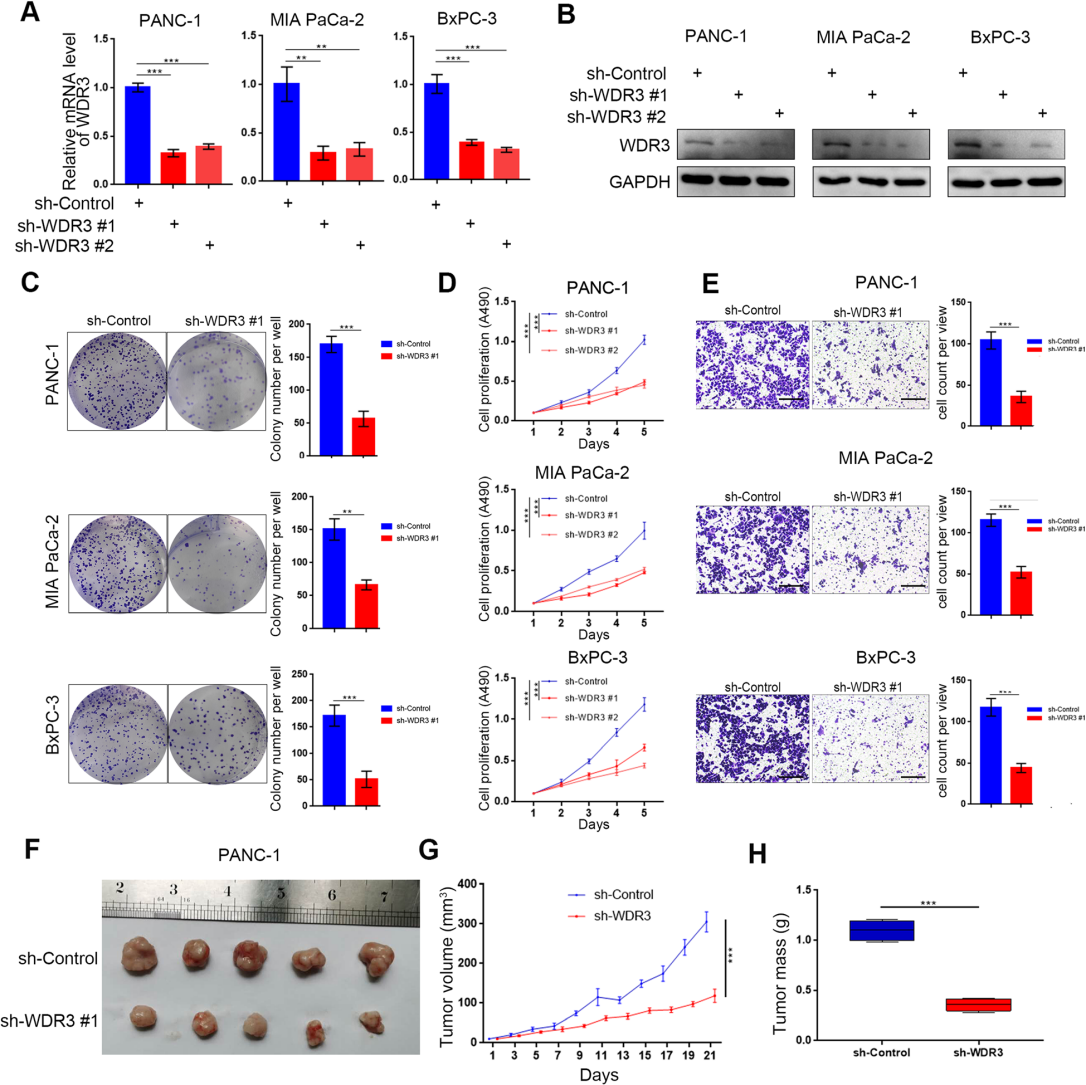
Silencing WDR3 suppresses the aggressive behavior of pancreatic cancer cells in vitro and pancreatic tumor growth in vivo*.* **a** and **b** RT-PCR (**a**) and western blot analyses (**b**) of WDR3 expression in PANC-1, MIA PaCa-2, and BxPC-3 cells infected with sh-Control or sh-WDR3s. GAPDH served as an internal reference. Data are presented as the mean ± SD of three independent experiments. Each sh-WDR3 group was compared with sh-Control group. Statistical analyses were performed with one-way ANOVA followed by Tukey’s multiple comparison’s tests. **, *P* < 0.01; ***, *P* < 0.001. **C-E.** PANC-1, MIA PaCa-2, and BxPC-3 cells were infected with sh-Control or sh-WDR3 #1. The cells were harvested for colony formation (**c**), MTS (**d**), and Transwell invasion assays (**e**) after 48 h of culture. Each bar represents the mean ± SD of three independent experiments. **, *P* < 0.01; ***, *P* < 0.001. **F-H.** PANC-1 cells infected with sh-Control or sh-WDR3 #1 were subcutaneously injected into nude mice. The tumors were harvested and photographed (**f**) on day 21. Data for tumor volume (**g**) and tumor mass (**h**) are shown as the mean ± SD (*n* = 5). Each sh-WDR3 group was compared with sh-Control group. Statistical analyses were performed with one-way ANOVA followed by Tukey’s multiple comparison’s tests. ***, *P* < 0.001

To explore the biological role of WDR3 in pancreatic cancer, pancreatic cancer cell lines with WDR3 silencing were established (Fig. 2a-b). Colony formation and MTS assays showed that WDR3 inhibition significantly inhibited the proliferation of pancreatic cancer cells (Fig. 2c-d), while a transwell invasion assay proved that WDR3 inhibition decreased the invasive ability of pancreatic cancer cells (Fig. 2e). To investigate the biological role of WDR3 in pancreatic cancer in vivo, PANC-1 cells with normal WDR3 expression or silenced WDR3 expression were subcutaneously injected into the left flank of nude mice under the same conditions for a xenograft assay. As Fig. 2f-h shows, the tumors formed by WDR3-silenced PANC-1 cells were smaller and lighter than those formed by WDR3 normal-expressing cells. Taken together, our results indicated that silenced WDR3 significantly inhibited the proliferation and invasion ability of pancreatic cancer cells in vitro and in vivo.

### Overexpressed WDR3 promoted the proliferation and invasion of pancreatic cancer cells in vitro and in vivo

**Fig. 3:**
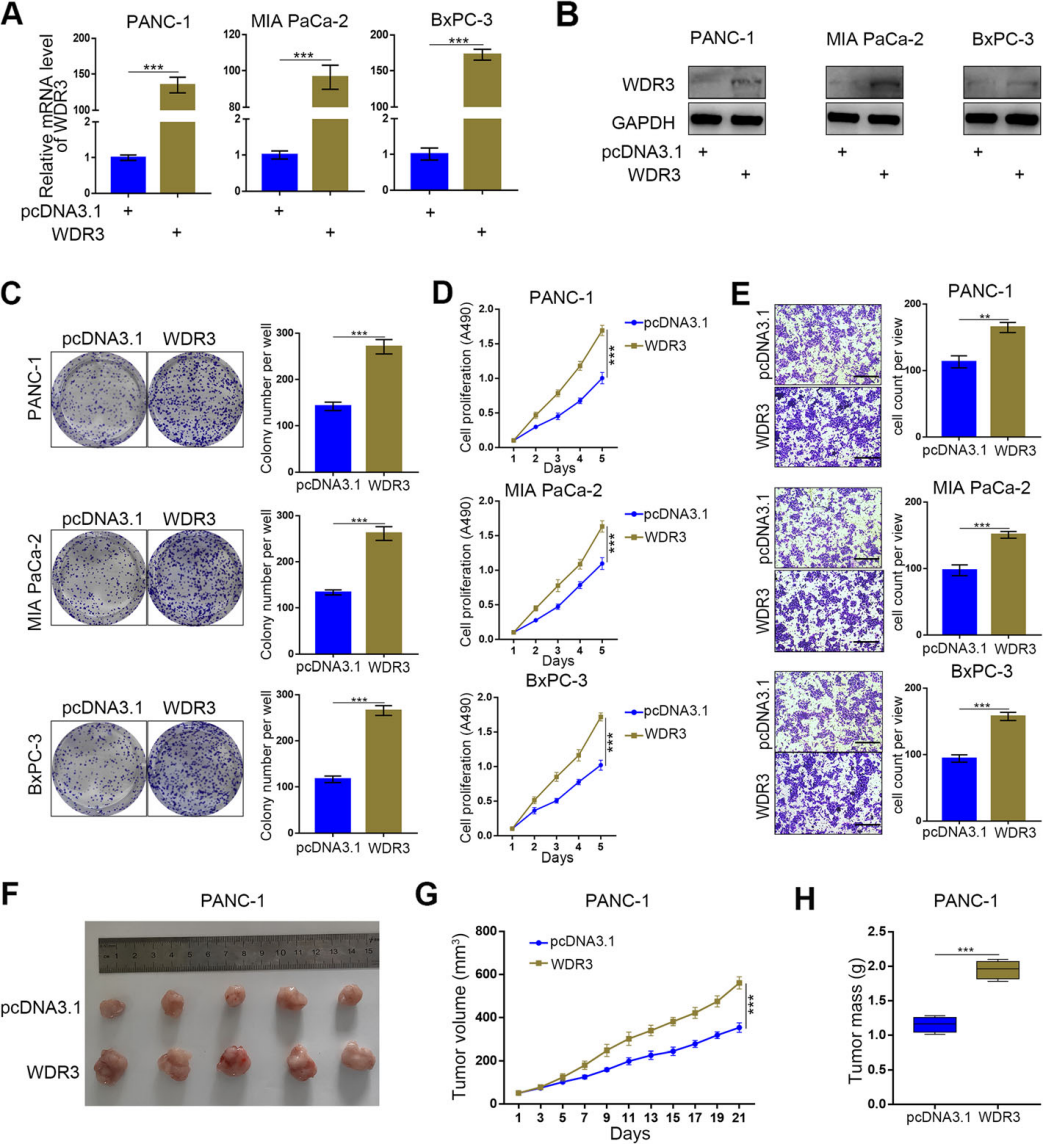
Overexpressed WDR3 promoted the aggressive behavior of pancreatic cancer cells in vitro and pancreatic tumor growth in vivo*.* **a** and **b** RT-PCR (**a**) and western blot analyses (**b**) of WDR3 expression in PANC-1, MIA PaCa-2, and BxPC-3 cells infected with pcDNA3.1 or WDR3 plasmid. GAPDH served as an internal reference. Data are presented as the mean ± SD of three independent experiments. WDR3 group was compared with pcDNA3.1 group. Statistical analyses were performed with one-way ANOVA followed by Tukey’s multiple comparison’s tests. ***, *P* < 0.001. **C-E.** PANC-1, MIA PaCa-2, and BxPC-3 cells were infected with pcDNA3.1 or WDR3 plasmid. The cells were harvested for colony formation (**c**), MTS (**d**), and Transwell invasion assays (**e**) after 48 h of culture. Each bar represents the mean ± SD of three independent experiments. **, *P* < 0.01; ***, *P* < 0.001. **F-H.** PANC-1 cells infected with pcDNA3.1 or WDR3 plasmid were subcutaneously injected into nude mice. The tumors were harvested and photographed (**f**) on day 21. Data for tumor volume (**g**) and tumor mass (**h**) are shown as the mean ± SD (*n* = 5). WDR3 group was compared with pcDNA3.1 group. Statistical analyses were performed with one-way ANOVA followed by Tukey’s multiple comparison’s tests. ***, *P* < 0.001

To further explore the biological role of overexpressed WDR3 in pancreatic cancer, pancreatic cancer cell lines with WDR3 overexpression were established (Fig. 3a-b). Colony formation and MTS assays showed that WDR3 overexpression significantly promoted the proliferation of pancreatic cancer cells (Fig. 3c-d), while a transwell invasion assay proved that WDR3 overexpression increased the invasive ability of pancreatic cancer cells (Fig. 3e). To investigate the biological role of overexpressed WDR3 in pancreatic cancer in vivo, PANC-1 cells with normal WDR3 expression or overexpressed WDR3 expression were subcutaneously injected into the left flank of nude mice under the same conditions for a xenograft assay. As Fig. 3f-h shows, the tumors formed by WDR3-overexpressed PANC-1 cells were larger and heavier than those formed by WDR3 normal-expressing cells. Taken together, our results indicated that overexpressed WDR3 significantly promoted the proliferation and invasion ability of pancreatic cancer cells in vitro and in vivo.

### Overexpressed WDR3 induced the activation of the hippo pathway in pancreatic cancer

**Fig. 4:**
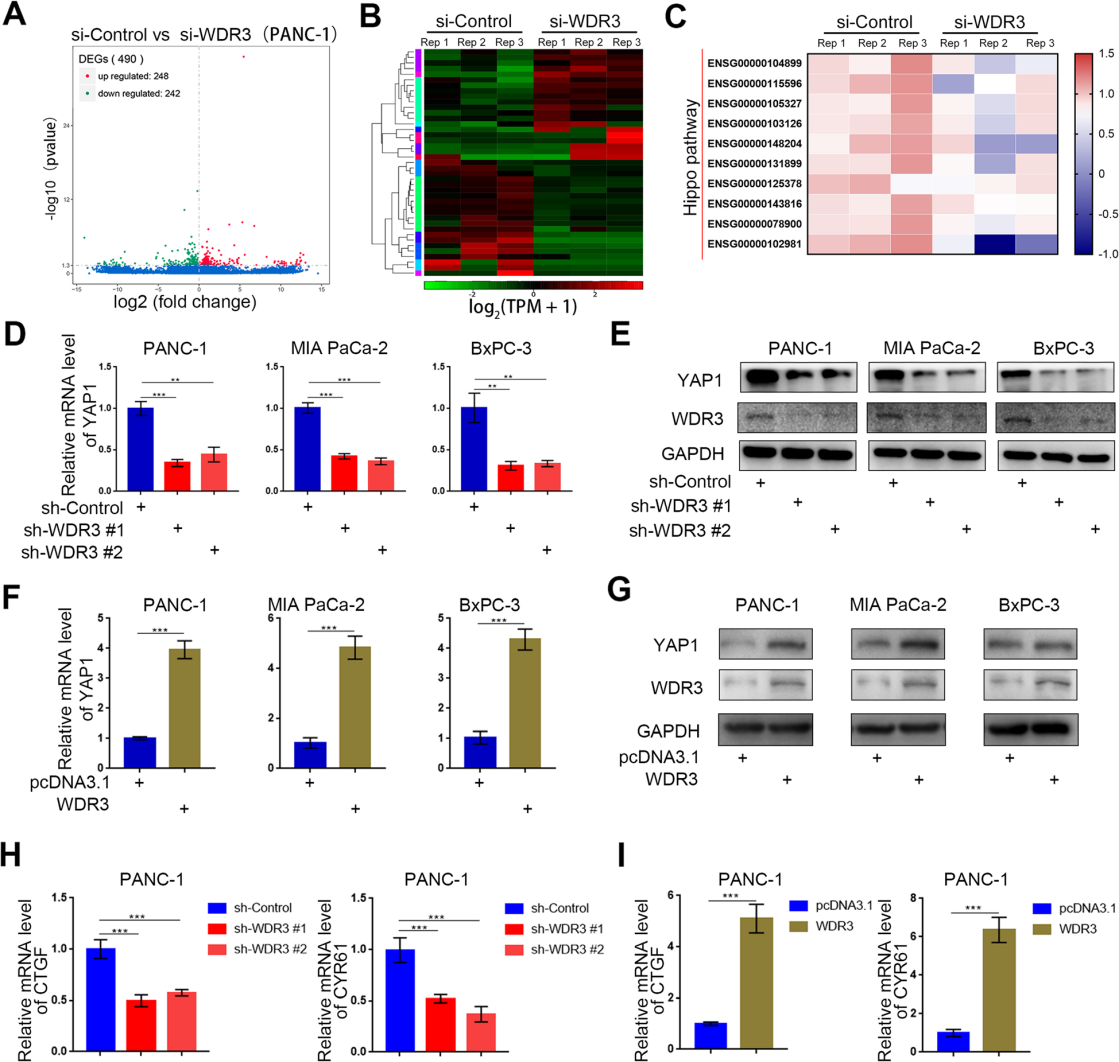
WDR3 transcriptionally increases YAP1 expression in pancreatic cancer cells. **a** and **b**. Volcano plot (**a**) and heatmap (**b**) showing the differentially expressed genes in PANC-1 cells infected with si-Control or si-WDR3. The blue points represent the downregulated genes (*n* = 248), while the red points represent the upregulated genes (*n* = 242). **c** Heatmap showing a subset of WDR3 knockdown-regulated genes with a *p*-value < 0.1 participating in the Hippo signaling pathway in PANC-1 cells. si-WDR3 group was compared with si-Control group. Statistical analyses were performed with one-way ANOVA followed by Tukey’s multiple comparison’s tests. **d** and **e** RT-PCR analysis (**d**) and western blot analysis (**e**) to detect the mRNA and protein expression levels of YAP1 in pancreatic cancer cells infected with sh-Control or sh-WDR3s. GAPDH served as an internal reference. Data are shown as the mean ± SD (*n* = 3). Statistical analyses were performed with one-way ANOVA followed by Tukey’s multiple comparison’s tests. **, *P* < 0.01; ***, *P* < 0.001. **F-G.** RT-PCR analysis (**f**) and western blot analysis (**g**) to detect the mRNA and protein expression levels of YAP1 in pancreatic cancer cells infected with pcDNA3.1 or WDR3. GAPDH served as an internal reference. Data are shown as the mean ± SD (*n* = 3). Statistical analyses were performed with one-way ANOVA followed by Tukey’s multiple comparison’s tests. ***, *P* < 0.001. **h** RT-PCR analysis to detect the mRNA expression levels of CTGF and CYR61 in pancreatic cancer cells infected with sh-Control or sh-WDR3s. GAPDH served as an internal reference. Data are shown as the mean ± SD (*n* = 3). Statistical analyses were performed with one-way ANOVA followed by Tukey’s multiple comparison’s tests. ***, *P* < 0.001. **i** RT-PCR analysis to detect the mRNA expression levels of CTGF and CYR61 in pancreatic cancer cells infected with pcDNA3.1 or WDR3. GAPDH served as an internal reference. Data are shown as the mean ± SD (*n* = 3). Statistical analyses were performed with one-way ANOVA followed by Tukey’s multiple comparison’s tests. ***, *P* < 0.001

We have proved that overexpressed WDR3 could increase the proliferation and invasion abilities of pancreatic cancer cells and was correlated with poor survival in pancreatic cancer patients. However, the specific regulatory mechanism underlying these effects is still unclear. By silencing WDR3 expression in PANC-1 cells and performing an RNA-seq assay, we identified 490 differentially expressed genes (DEGs), including 248 upregulated DEGs and 242 downregulated DEGs (Fig. 4a-b). Additionally, KEGG signaling pathway analysis showed that the Hippo signaling pathway was significantly inhibited after WDR3 silencing (Fig. 4c), which indicated that WDR3 could regulate the activation of the Hippo signaling pathway. Consistently, PCR and western blot analyses showed that both the mRNA and protein levels of YAP1, the main effector of the Hippo signaling pathway, were significantly downregulated in WDR3-silenced pancreatic cancer cells (Fig. 4d-e), while both the mRNA and protein levels of YAP1 were significantly upregulated in WDR3-overexpressing pancreatic cancer cells (Fig. 4f-g). Furthermore, the mRNA expression of CTGF and CYR61, the major downstream regulatory genes in the Hippo signaling pathway, was also positively correlated with the expression level of WDR3 in pancreatic cancer cells (Fig. 4h-i). Furthermore, by overexpressing YAP1 in WDR3 silenced PANC-1 cells, we found that overexpressed YAP1 reversed the inhibition of the proliferation and invasion ability in pancreatic cancer cells induced by WDR3 silencing (Supplementary Fig. 1). These results indicated that WDR3 promoted the proliferation and invasion ability of pancreatic cancer cells by transcriptionally upregulating YAP1 expression and activating the Hippo signaling pathway in pancreatic cancer cells.

### WDR3 protein expression positively correlated with YAP1 levels in cancer patient specimens

We demonstrated that WDR3 could regulate YAP1 expression in pancreatic cancer cells, but the clinical relationship between WDR3 and YAP1 in human pancreatic cancer specimens remains unclear. By conducting IHC analysis of a cohort of PDAC patients (*n* = 31), we found a positive correlation between the protein expression levels of WDR3 and YAP1 in PDAC specimens (Spearman correlation *r* = 0.5916, *P* < 0.001) (Supplementary Fig. 2A-C). Consistently, the GEPIA searching result showed a positive correlation between WDR3 and YAP1 mRNA expression levels in pancreatic cancer patient specimens (Supplementary Fig. 2D). Therefore, all these results suggested a positive correlation between the expression levels of WDR3 and YAP1 in pancreatic cancer specimens.

### The WDR3 knockdown enhanced the anti-pancreatic cancer effect of YAP1 inhibition

**Fig. 5:**
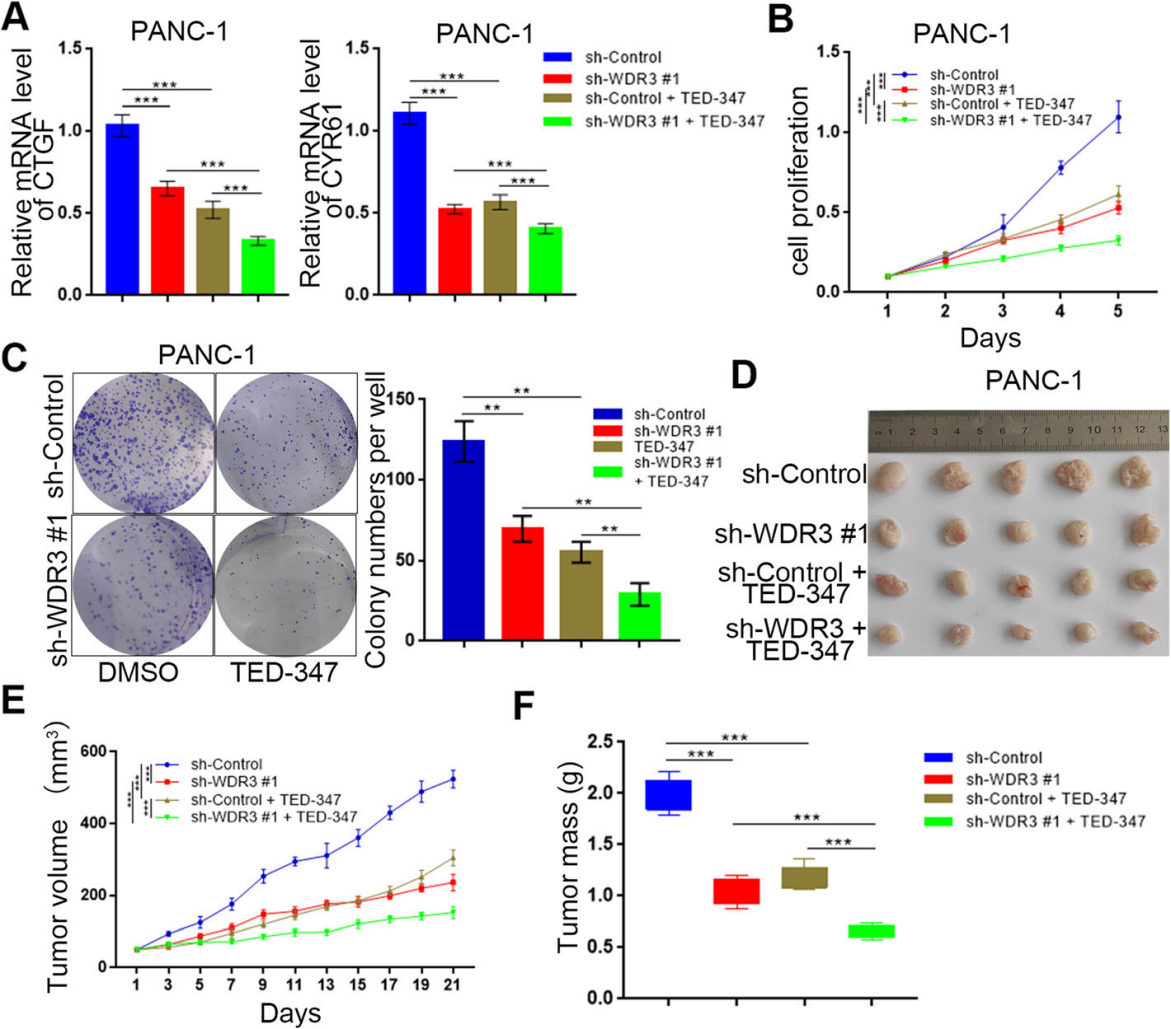
WDR3 knockdown enhanced the anti-pancreatic cancer effect of YAP1 inhibition. **a** RT-PCR analysis was used to detect the mRNA expression levels of CTGF and CYR61 in pancreatic cancer cells infected with sh-Control or sh-WDR3 #1 and treated with or without TED-347. GAPDH served as an internal reference. Data are shown as the mean ± SD (*n* = 3). Statistical analyses were performed with one-way ANOVA followed by Tukey’s multiple comparison’s tests. ***, *P* < 0.001. **b**-**c.** PANC-1 cells infected with sh-Control or sh-WDR3 #1 and treated with or without TED-347 were harvested for MTS (**b**) and colony formation assays (**c**). Data are shown as the mean ± SD (*n* = 3). Statistical analyses were performed with one-way ANOVA followed by Tukey’s multiple comparison’s tests. ***, *P* < 0.001. **d**-**f.** PANC-1 cells infected with sh-Control or sh-WDR3 #1 were subcutaneously injected into nude mice. The mice were treated with TED-347 3 times on days 1, 4, and 7 at a dose of 20 mg/kg. The tumors were harvested and photographed (**d**) on day 21. Data for tumor volume (**e**) and tumor mass (**f**) are shown as the mean ± SD (*n* = 5). Statistical analyses were performed with two-way ANOVA followed by Sidak’s multiple comparison’s tests. ***, *P* < 0.001

TED-347 is a selective inhibitor of YAP1 at the protein level that functions by inhibiting the binding of TEAD4 to full-length Yap1 in a dose- and time-dependent manner [24]. Since WDR3 silencing could inhibit YAP1 expression at the mRNA and protein levels, we hypothesized that The WDR3 knockdown enhanced the anti-pancreatic cancer effect of YAP1 inhibition. RT-PCR analysis showed an enhanced effect for WDR3 silencing and TED-347 treatment, resulting in decreased levels of the downstream target genes of YAP1, CTGF and CYR61 (Fig. 5a). Besides, MTS and colony formation assays indicated the synergistic effect of WDR3 silencing and TED-347 treatment, demonstrating inhibition of the proliferative ability of pancreatic cancer cells in vitro (Fig. 5b-c). Furthermore, by subcutaneously injecting normal WDR3-expressing or WDR3-silenced PANC-1 cells into the left flank of nude mice under the same conditions for a xenograft assay and treating the mice with or without TED-347, we found that WDR3 silencing and TED-347 treatment could both slow tumor growth, and the combined treatment group showed further inhibition of tumor growth (Fig. 5d-f). Consistently, the orthotopic syngeneic model of pancreatic cancer in C57BL/6 mice verified that the combined treatment of WDR3 silencing and TED-347 treatment further inhibited of tumor growth of pancreatic cancer (Supplementary Fig. 3). In brief, all these data showed that WDR3 knockdown enhanced the anti-pancreatic cancer effect of YAP1 inhibition both in vitro and in vivo.

### The regulation of YAP1 induced by WDR3 was dependent on GATA4 in pancreatic cancer cells

**Fig. 6:**
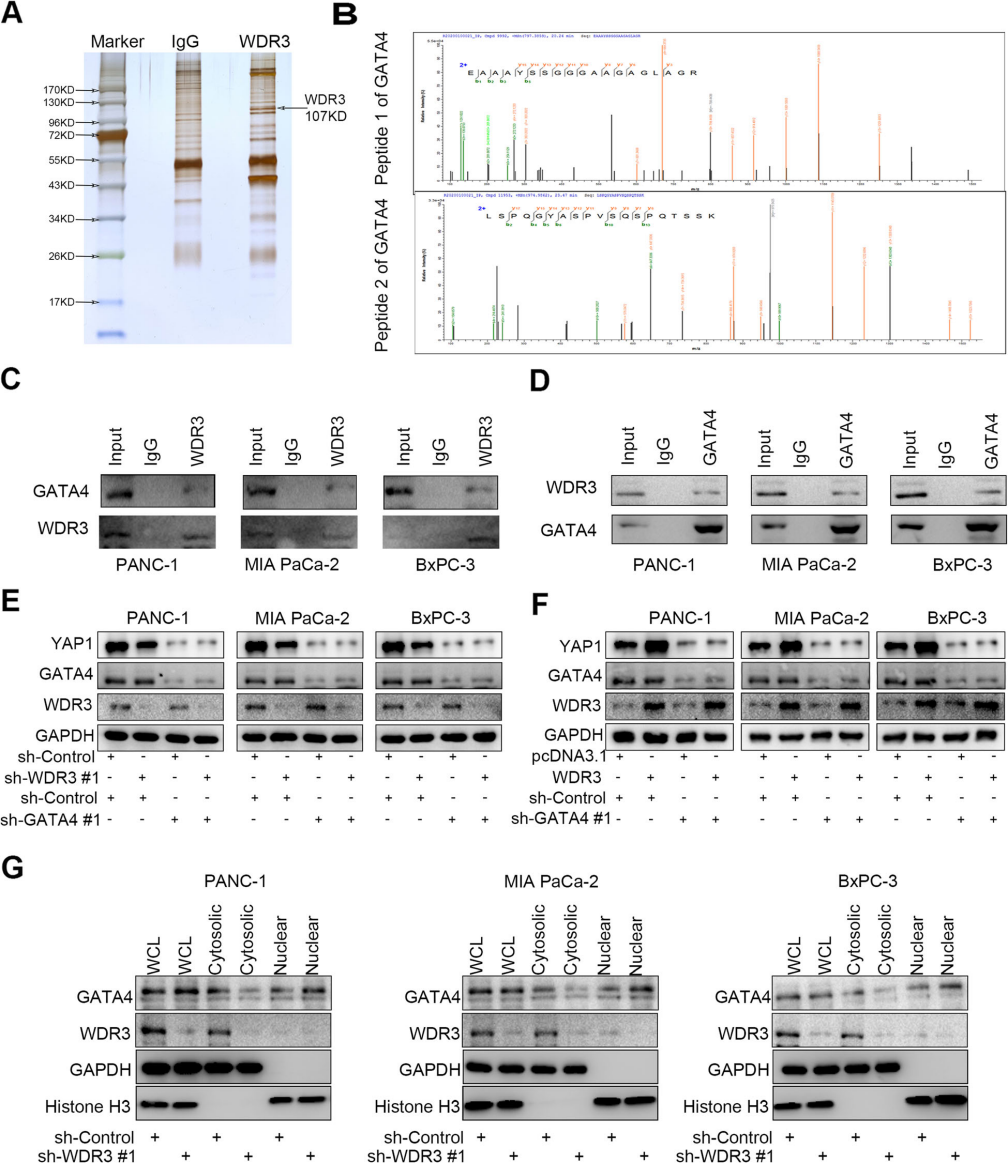
The regulation of YAP1 induced by WDR3 was dependent on GATA4 in pancreatic cancer cells. A-B. LC-MS/MS identified an interaction between WDR3 and GATA4 (**a**) by detecting two peptides of GATA4 (**b**). **c**-**d** Coimmunoprecipitation showed the interaction between WDR3 and GATA4. **e**-**f** Western blot analysis showed the protein expression levels of specific genes. GAPDH served as an internal reference. **g** Western blot analysis to show the GATA4 expression in the nucleus and cytoplasm of pancreatic cancer cells infected with sh-Control or sh-WDR3

As mentioned above, WDR3 silencing could inactivate the Hippo signaling pathway by decreasing YAP1 expression in pancreatic cancer cells. However, the specific mechanism remained unclear. We performed immunoprecipitation and LC-MS/MS assays to identify WDR3-associated proteins, and GATA4 was identified as a potential WDR3-interacting protein (Fig. 6a-b and Supplementary Fig. 4), which was verified by western blot analysis of immunoprecipitated samples (Fig. 6c-d). Then, we hypothesized that the regulation of YAP1 by WDR3 is dependent on the interaction with GATA4. GATA4 knockdown reversed not only the inhibition of YAP1 induced by WDR3 silencing (Fig. 6e) but also the upregulation of YAP1 expression induced by WDR3 overexpression (Fig. 6f). Consistently, GATA4 knockdown also reversed the inhibition of proliferation and invasion ability of pancreatic cancer cells induced by WDR3 silencing (Supplementary Fig. 5). Furthermore, WDR3 silencing couldn’t regulate the total expression level of GATA4, whereas could increase the intranuclear expression level of GATA4 (Fig. 6g). Therefore, the regulation of YAP1 by WDR3 was found to be dependent on GATA4 in pancreatic cancer cells.

### GATA4, acting as a transcription factor, transcriptionally upregulated YAP1 expression in pancreatic cancer cells

**Fig. 7:**
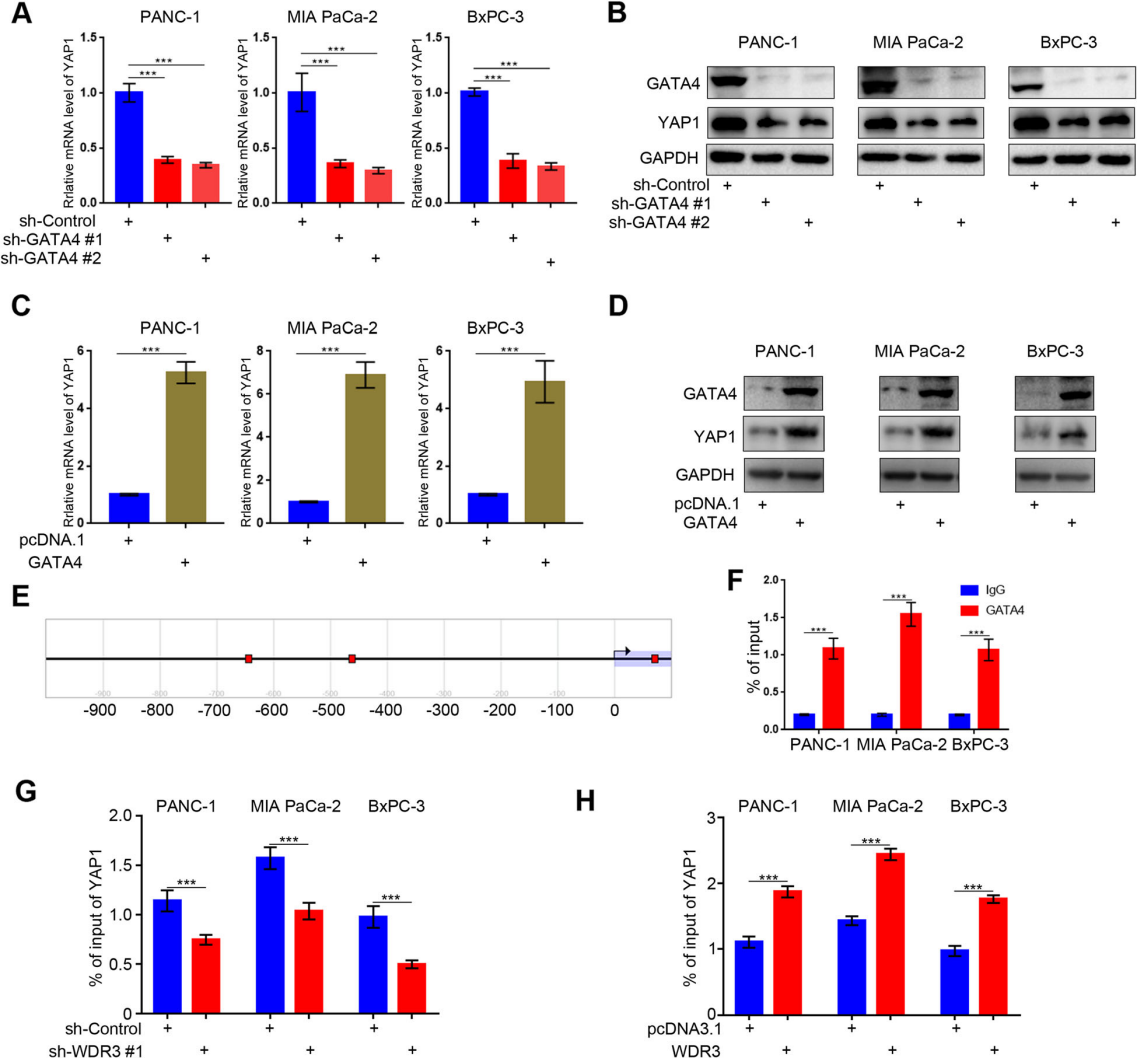
GATA4, acting as a transcription factor, transcriptionally upregulated YAP1 expression in pancreatic cancer cells. **a**-**b** RT-PCR analysis (**a**) and western blot analysis (**b**) were used to detect the mRNA and protein expression levels of YAP1 in pancreatic cancer cells infected with sh-Control or sh-GATA4s. GAPDH served as an internal reference. Data are shown as the mean ± SD (*n* = 3). Statistical analyses were performed with two-way ANOVA followed by Sidak’s multiple comparison’s tests. ***, *P* < 0.001. **C-D.** RT-PCR analysis (**c**) and western blot analysis (**d**) were used to detect the mRNA and protein expression levels of YAP1 in pancreatic cancer cells infected with pcDNA3.1 or GATA4.GAPDH served as an internal reference. Data are shown as the mean ± SD (*n* = 3). Statistical analyses were performed with two-way ANOVA followed by Sidak’s multiple comparison’s tests. ***, *P* < 0.001. **e** The Eukaryotic Promoter Database was searched to evaluate potential YAP1 promoter binding by GATA4 (− 649 bp, − 467 bp, and 66 bp), and the ChIP primer sequences (Table S3) were designed for the gene locus from − 700 bp to − 400 bp. **f** GATA4 ChIP-qPCR of YAP1 in PANC-1, MIA PaCa-2, and BxPC-3 cells were performed. All data are shown as the mean ± SD of three replicates. Statistical analyses were performed with two-way ANOVA followed by Sidak’s multiple comparison’s tests. ***, *P* < 0.001. **G-H.** GATA4 ChIP-qPCR of YAP1 in PANC-1, MIA PaCa-2, and BxPC-3 cells with WDR3 silenced (**g**) or overexpressed (**h**). was performed. All data are shown as the mean ± SD of three replicates. Statistical analyses were performed with two-way ANOVA followed by Sidak’s multiple comparison’s tests. ***, *P* < 0.001

As a transcription factor, GATA4 can regulate the expression of numerous tumor-related genes in pancreatic cancer [25]. Since the regulation of YAP1 by WDR3 was found to be dependent on GATA4 (Fig. 6), we speculated that GATA4 can transcriptionally regulate YAP1 expression as a transcription factor. Consistently, RT-PCR and western blot assays indicated that GATA4 knockdown could significantly inhibit YAP1 expression at both the mRNA and protein levels (Fig. 7a-b), while GATA4 overexpression could significantly induce YAP1 expression at both the mRNA and protein levels (Fig. 7c-d). More importantly, ChIP-qPCR analysis identified that GATA4 could bind to the promoter of the YAP1 gene (Fig. 7e-f), which could be inhibited by WDR3 silencing (Fig. 7g) and induced by WDR3 overexpression (Fig. 7h). In conclusion, we proved that GATA4, acting as a transcription factor, could transcriptionally upregulate YAP1 expression in pancreatic cancer cells.

## Discussion

In our study, we first proved that WDR3 was overexpressed and positively correlated with poor survival in pancreatic cancer (Fig. 1). Akdi et al reported that WDR3 is overexpressed and a risk factor in thyroid cancer [9]. Additionally, several groups have also reported the biological role of WDR3 in modulating genome stability [10], increasing cancer predisposition [26], promoting proliferation and arresting cancer cells in the G1 phase of the cell cycle [7]. Consistently, our results also indicated that overexpressed WDR3 increased the proliferation and invasion abilities of pancreatic cancer cells (Fig. 2). However, the biological mechanism of WDR3 overexpression in pancreatic cancer still needs further study.

GATA binding protein 4 (GATA4), a protein in the GATA family of zinc-finger transcription factors, can recognize the GATA motif, which is present in the promoter of many tumor-related genes. GATA4 regulates the expression of genes involved in multiple pathological/physiological processes, including embryogenesis, myocardial differentiation and function, and normal testicular development. It has been reported GATA4 expression is associated with increased tumor size, metastasis, and a poor prognosis [27]. GATA4 mRNA expression is upregulated in pancreatic cancer cell lines and tissues [25], and downregulation of GATA4 expression increases drug sensitivity in cancer cells [28]. GATA4 can decrease P53 protein expression by transcriptionally activating the expression of MDM2 [29], the primary negative regulatory factor of the P53 protein that induces p53 ubiquitination and degradation [30]. GATA4 is also highly expressed in most hepatoblastomas and correlates with a mesenchymal, migratory phenotype in hepatoblastoma cells by regulating the expression of ADD3, AHNAK, and IGFBP1 [31]. Similarly, our results indicated that GATA4 could function as a transcription factor to induce YAP1 expression and activate the Hippo signaling pathway, which resulted in pancreatic cancer progression. GATA4 knockdown reversed the inhibition of YAP1 and proliferation and invasion of abilities of pancreatic cancer cells induced by WDR3 silencing and reversed the upregulation of YAP1 expression induced by WDR3 overexpression (Fig. 6). Taken together, our results provide new insights into the specific mechanism by which GATA4 regulates the progression of pancreatic cancer.

The Hippo signaling pathway was first discovered in studies of *Drosophila melanogaster* [32]. Hippo signaling governs normal organ development and tissue regeneration under physiological conditions [33]. Hippo signaling is an evolutionarily conserved network that plays a key role in regulating cell proliferation, organ growth, and regeneration [34]. YAP1 is the key downstream regulator in the Hippo pathway that exhibits upregulated expression in pancreatic cancer [35–37]. Aberrant transcriptional activity of YAP1 has crucial roles in pancreatic tumor cell biology, including roles in growth, epithelial-mesenchymal transition (EMT), microenvironmental signaling transduction, and drug resistance [34]. Then, inhibition of YAP1 expression is essential for pancreatic cancer targeted therapy. Studies have shown that YAP1 expression is induced by KRAS activation [35], aerobic glycolysis [38], GNAS [39], and the cancer upregulated gene (CUG) 2 exhibiting upregulated expression in lung cancer which could increase the expression of YAP1 [40]. Interestingly, our results identified a protein interaction between WDR3 and GATA4 that led to the regulation of GATA4 nuclear translocation and YAP1 expression in pancreatic cancer. Silencing WDR3 significantly decreased the expression levels of YAP1 and the downstream target genes CTGF and CYR61 in pancreatic cancer. All these results emphasized the clinical significance of WDR3-targeted therapy.

TED-347 is a potent, irreversible, covalent, allosteric inhibitor of the YAP-TEAD protein-protein interaction [24]. TED-347 forms a covalent complex with TEAD4 that inhibits TEAD4 binding to YAP1, blocks YAP1 transcriptional activity, and suppresses the expression of downstream target genes, including CTGF and CYR61. Combined with the inhibition of YAP1 transcription induced by WDR3 knockdown, TED-347 treatment further enhanced the ability of WDR3 silencing to inhibit pancreatic cancer progression.

## Conclusions

**Fig. 8:**
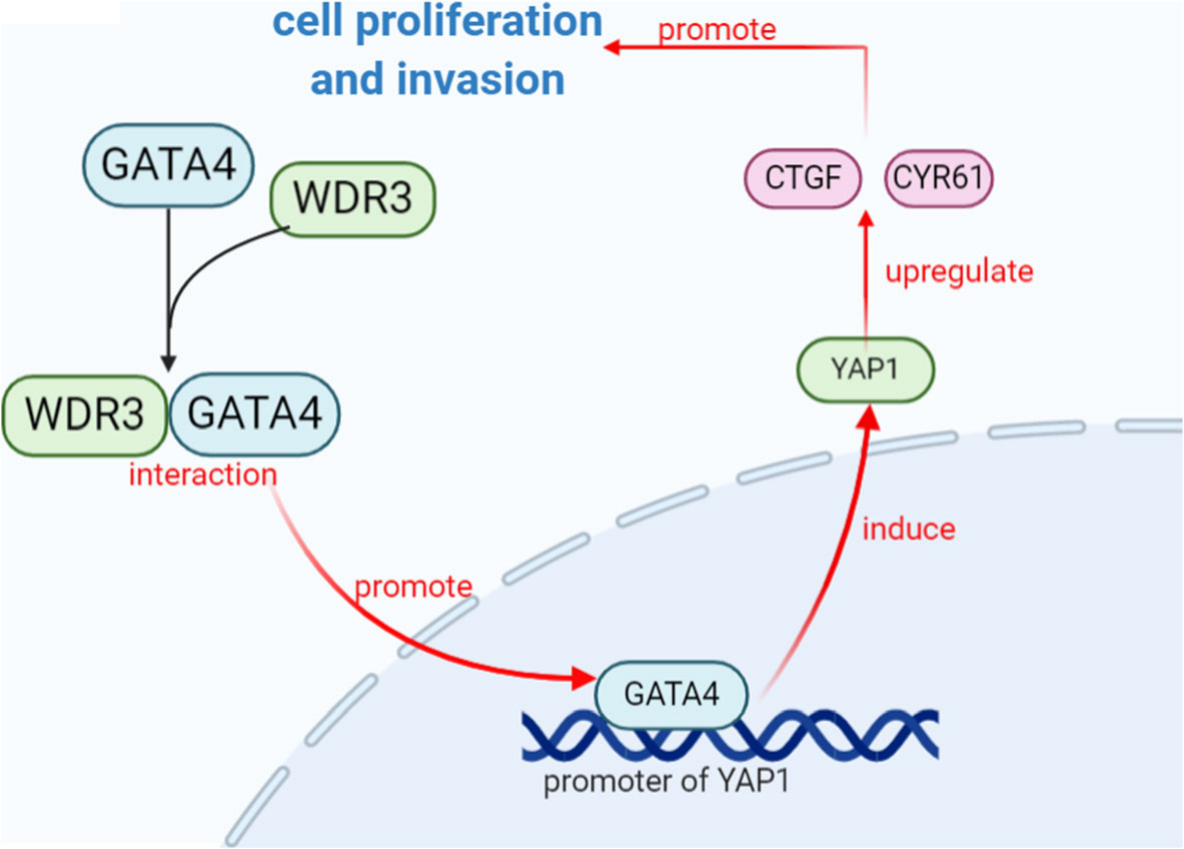
Overexpressed WDR3 induces the activation of Hippo pathway by interacting with GATA4 in pancreatic cancer. The overexpressed WDR3 promoted the nuclear translocation of GATA4 and increased the binding of GATA4 to the promoter of YAP1 by interacting with GATA4 in pancreatic cancer cells. As a transcription factor, the binding of GATA4 to the promoter of YAP1 induced the expression of YAP1. The upregulation of YAP1, the main effector of the Hippo signaling pathway, resulted in the activation of the Hippo pathway signaling and the overexpression of downstream effector genes, CTGF and CYR61. Finally, the cell proliferation and invasion were promoted

Our study proved that overexpressed WDR3 was correlated with poor survival in pancreatic cancer and WDR3 silencing significantly inhibited the proliferation, invasion, and tumor growth of pancreatic cancer. Furthermore, WDR3 induced YAP1 expression by interacting with GATA4 and inducing the nuclear translocation of GATA4, the transcription factor of YAP1, in pancreatic cancer cells. Finally, the combination of WDR3 silencing and administration of the YAP1 inhibitor TED-347 had a synergistic inhibitory effect on the progression of pancreatic cancer (Fig. 8). Therefore, WDR3 is potentially a therapeutic target for pancreatic cancer treatment.

## Supplementary Information

**Additional file 1: Fig. S1.** YAP1 overexpression reversed the inhibition in the aggressive behavior of pancreatic cancer cells induced by WDR3 silencing in vitro*.* **Fig. S2.** WDR3 expression was positively correlated with YAP1 expression in pancreatic cancer patient specimens. **Fig. S3.** WDR3 silencing enhanced the anti-pancreatic cancer effect of TED-347 treatment in the orthotopic syngeneic model of pancreatic cancer. **Fig. S4.** LC-MS/MS assay to identify the base peak of IgG IP and WDR3 IP samples. **Fig. S5.** GATA4 silencing reversed the induction in the aggressive behavior of pancreatic cancer cells induced by WDR3 overexpression in vitro*.* **Table S1.** The primer sequences for RT-qPCR. **Table S2.** The shRNA sequences. **Table S3.** The primer sequences for ChIP-qPCR.

## Data Availability

The datasets during and/or analyzed during the current study available from the corresponding author on reasonable request. Gene correlation analyses between the mRNA expression levels of WDR3 and YAP1 were carried out with the GEPIA database (http://gepia.cancer-pku.cn/) for all given sets of GTEx and TCGA expression data. The Eukaryotic Promoter Database (https://epd.epfl.ch//index.php) was used to determine the potential binding sites of GATA4 in the promoter of the YAP1 gene.

## Abbreviations

PDAC: Pancreatic ductal adenocarcinoma
WDR3: WD repeat domain 3
YAP1: Yes association protein 1
GATA4: GATA binding protein 4
TEAD: TEA domain
CTGF: Connective tissue growth factor
CYR61: Cysteine rich angiogenic inducer 61
KEGG: Kyoto Encyclopedia of Genes and Genomes
TCGA: The Cancer Genome Atlas
COAD: Colon adenocarcinoma
ESCA: Esophageal carcinoma
LIHC: Liver hepatocellular carcinoma
PAAD: Pancreatic adenocarcinoma
STAD: Stomach adenocarcinoma
DFS: Disease-free survival
OS: Overall survival
