# Supplementary material for: Overexpressed WDR3 induces the activation of Hippo pathway by interacting with GATA4 in pancreatic cancer
Source: J Exp Clin Cancer Res. 2021 Mar 1;40:88. doi: 10.1186/s13046-021-01879-w (PMC7923337; doi:10.1186/s13046-021-01879-w)
Supplement: Supplementary file 1 — Additional file 1: Fig. S1. YAP1 overexpression reversed the inhibition in the aggressive behavior of pancreatic cancer cells induced by WDR3 silencing in vitro. Fig. S2. WDR3 expression was positively correlated with YAP1 expression in pancreatic cancer patient specimens. Fig. S3. WDR3 silencing enhanced the anti-pancreatic cancer effect of TED-347 treatment in the orthotopic syngeneic model of pancreatic cancer. Fig. S4. LC-MS/MS assay to identify the base peak of IgG IP and WDR3 IP samples. Fig. S5. GATA4 silencing reversed the induction in the aggressive behavior of pancreatic cancer cells induced by WDR3 overexpression in vitro. Table S1. The primer sequences for RT-qPCR. Table S2. The shRNA sequences. Table S3. The primer sequences for ChIP-qPCR. [file 13046_2021_1879_MOESM1_ESM.docx]

**Supplementary Data**

**Fig. S1. YAP1 overexpression reversed the inhibition in the aggressive behavior of pancreatic cancer cells induced by WDR3 silencing *in vitro***


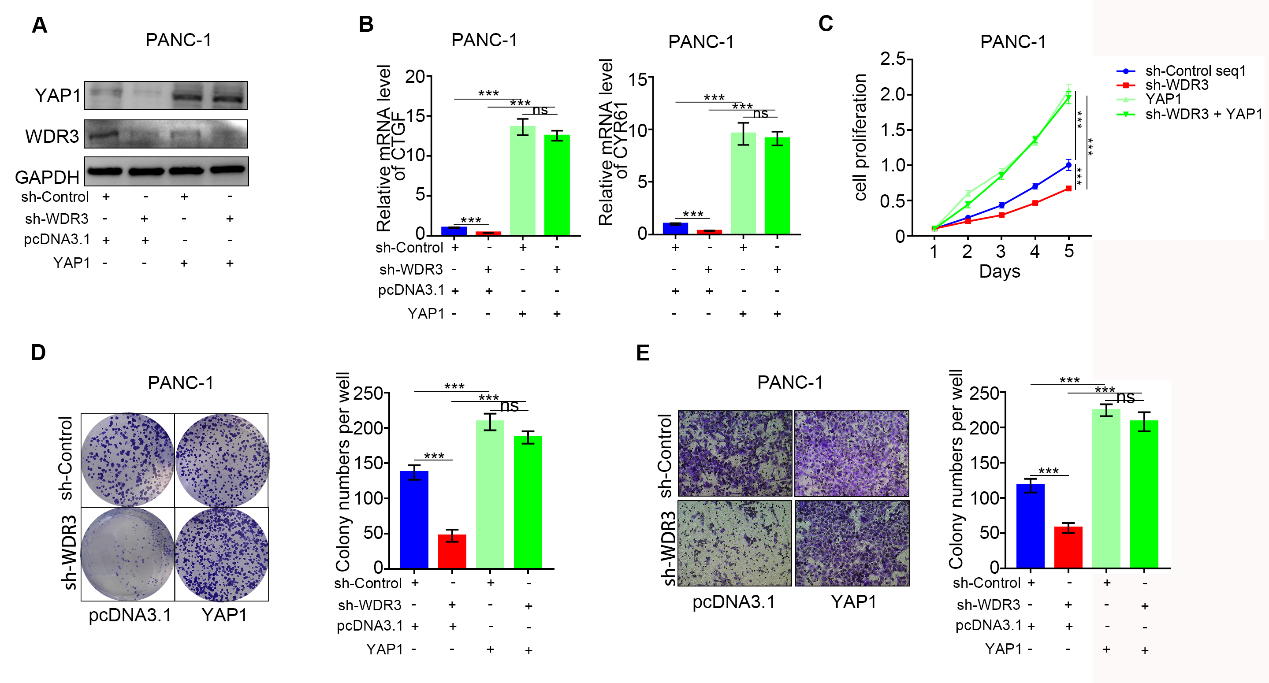


**A.** Western blot analyses of WDR3 and YAP1 expression in PANC-1 cells infected with sh-Control or sh-WDR3s and/or YAP1. GAPDH served as an internal reference.

**B.** RT-PCR analyses (B) of CTGF and CYR61 expression in PANC-1 cells infected with sh-Control or sh-WDR3s and/or YAP1. GAPDH served as an internal reference. Data are presented as the mean ± SD of three independent experiments. Statistical analyses were performed with one-way ANOVA followed by Tukey's multiple comparison's tests. ns, not significant; ***, P < 0.001.

**C-E.** PANC-1 infected with sh-Control or sh-WDR3s and/or YAP1 were were harvested for MTS (C), colony formation (D), and Transwell invasion assays (E) after forty-eight hours of culture. Each bar represents the mean ± SD of three independent experiments. Statistical analyses were performed with one-way ANOVA followed by Tukey's multiple comparison's tests. ns, not significant; ***, P < 0.001.

**Fig. S2. WDR3 expression was positively correlated with YAP1 in pancreatic cancer patient specimens.**


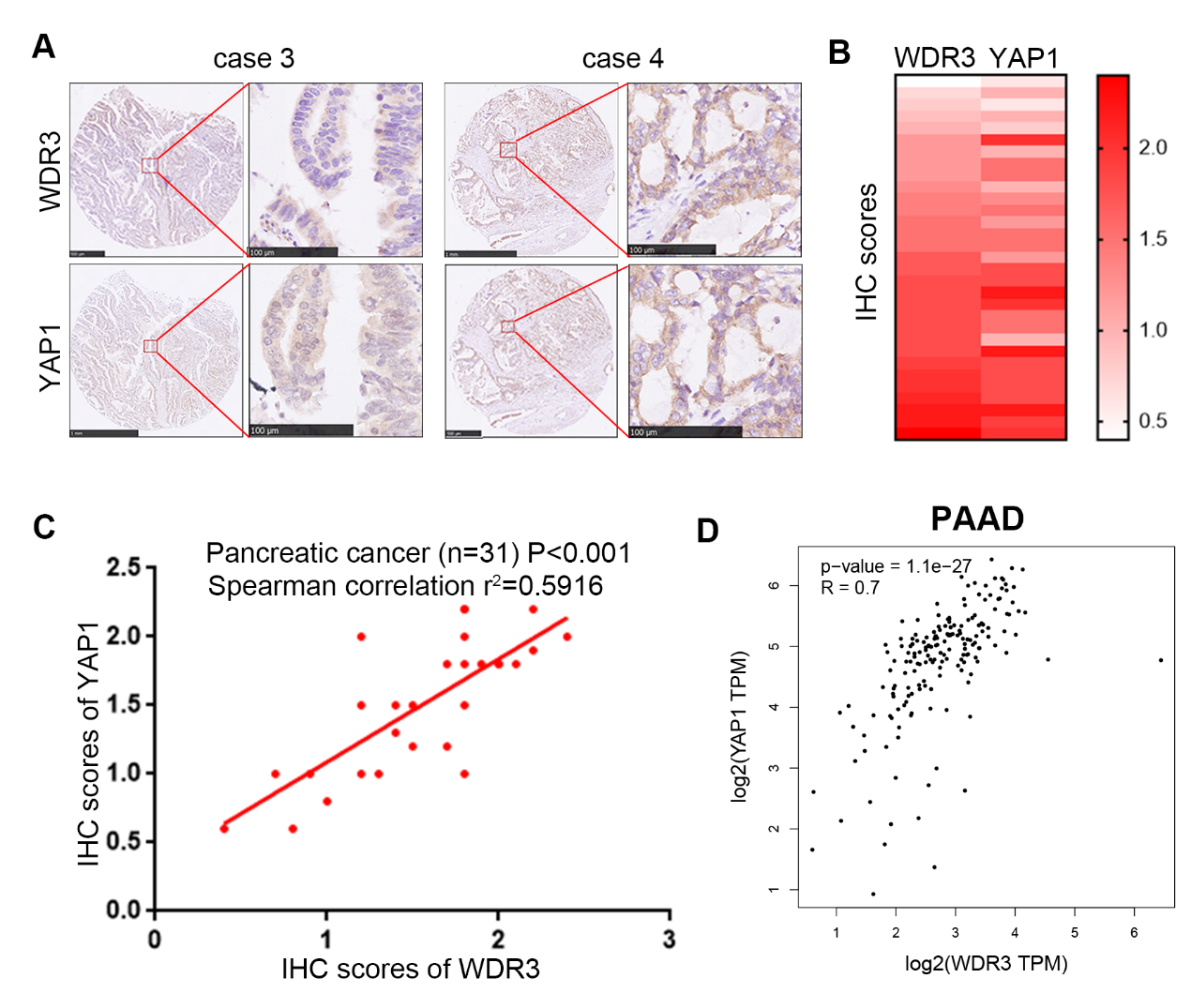


**A.** IHC images of WDR3 and YAP1 staining in TMA tissue sections (n = 31 PDAC samples). The scale bars are shown as indicated.

**B and C.** Heatmap (B) and dot plot (C) showing the correlation of IHC scores for the protein expression of WDR3 and YAP1 in PDAC patient specimens. (r = 0.5916 for Spearman correlation coefficients, P < 0.001).

**D.** Evaluation of the correlation between the expression of WDR3 and YAP1 at the mRNA level in pancreatic cancer samples performed with the GEPIA web tool (P = 1.1e-27, R = 0.7).

**Fig. S3. WDR3 knockdown enhanced the anti-pancreatic cancer effect of YAP1 inhibition.**


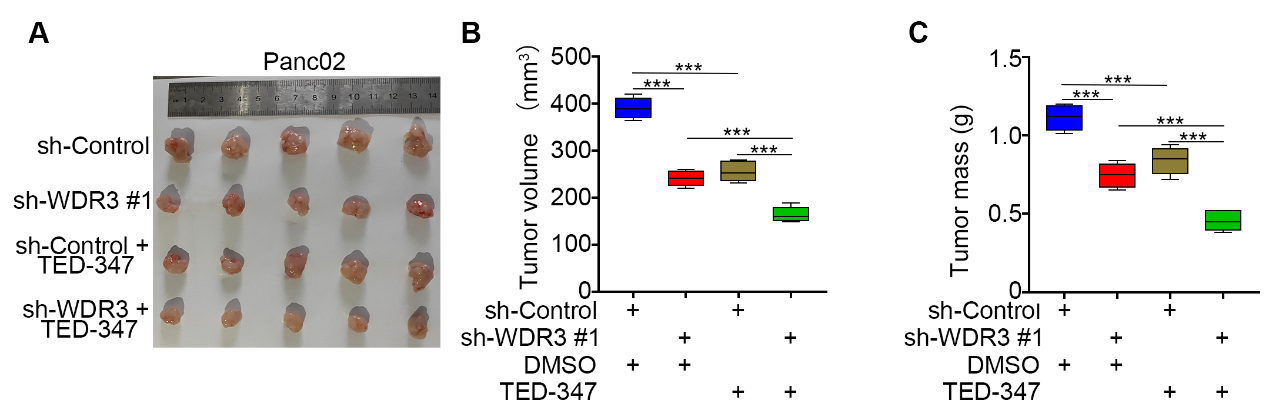


**A-C.** Panc02 cells infected with sh-Control or sh-WDR3 #1 were subcutaneously injected into C57BL/6 mice. The mice were treated with TED-347 3 times on days 1, 4 and 7 at a dose of 20 mg/kg. The tumors were harvested and photographed (A) on day 21. Data for tumor volume (B) and tumor mass (C) are shown as the mean ± SD (n = 5). Statistical analyses were performed with two-way ANOVA followed by Sidak's multiple comparison's tests. ***, P < 0.001.

**Fig. S4. LC-MS/MS assay to identify the base peak of IgG IP and WDR3 IP samples.**


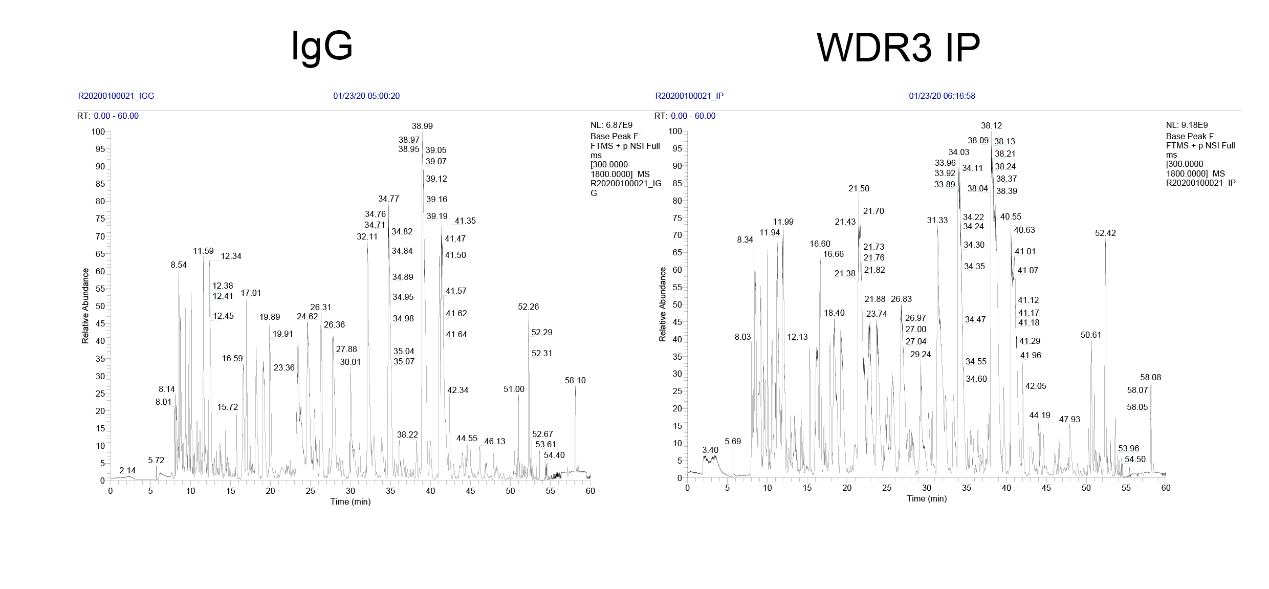


**Fig. S5. GATA4 silencing reversed the induction in the aggressive behavior of pancreatic cancer cells induced by WDR3 overexpression *in vitro***


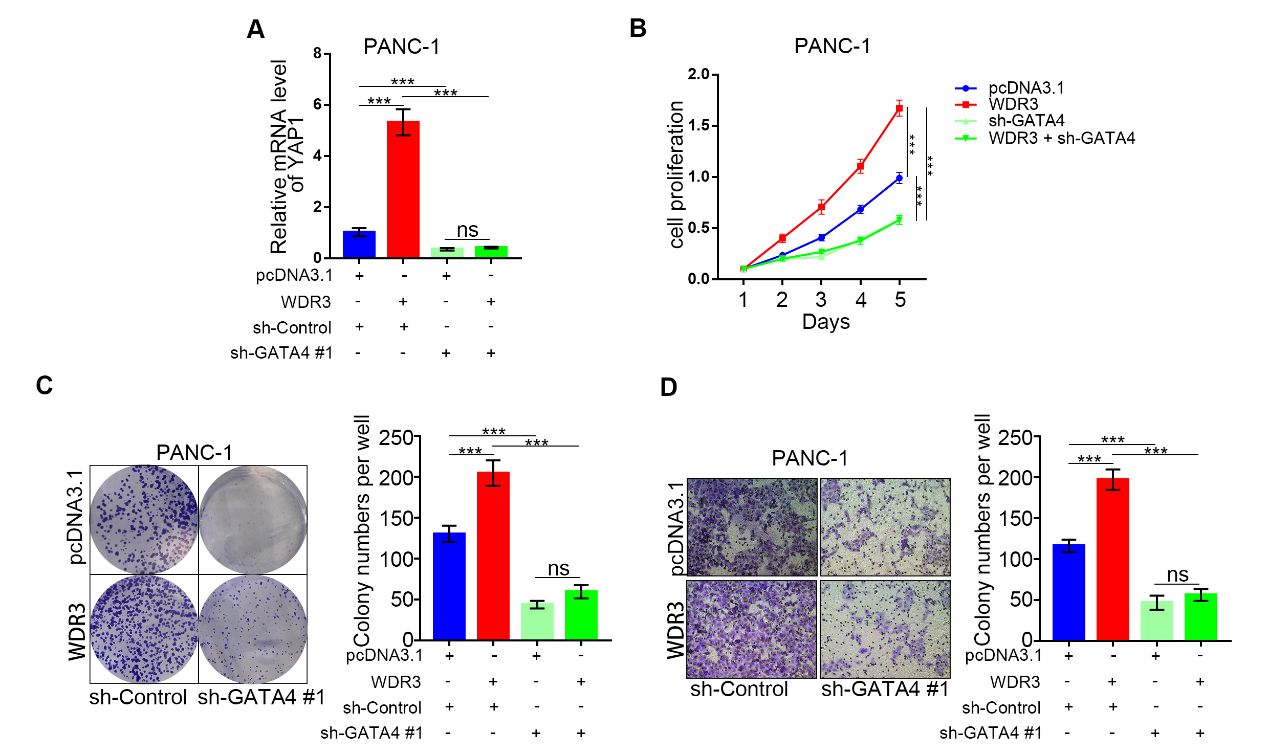


**A.** RT-PCR analyses (B) of YAP1 expression in PANC-1 cells infected with WDR3s and/or sh-GATA4 #1. GAPDH served as an internal reference. Data are presented as the mean ± SD of three independent experiments. Statistical analyses were performed with one-way ANOVA followed by Tukey's multiple comparison's tests. ns, not significant; ***, P < 0.001.

**B-D.** PANC-1 infected with WDR3s and/or sh-GATA4 #1 were were harvested for MTS (B), colony formation (C), and Transwell invasion assays (D) after forty-eight hours of culture. Each bar represents the mean ± SD of three independent experiments. Statistical analyses were performed with one-way ANOVA followed by Tukey's multiple comparison's tests. ns, not significant; ***, P < 0.001.

**Table S1. The primer sequences for RT-qPCR.**

| Gene | Forward primer (**5**′ - 3′) | Reverse primer (**5**′ - 3′) |
| --- | --- | --- |
| GAPDH | ATGACAATGAATACGGCTACAGCA | GCAGCGAACTTTATTGATGGTATT |
| WDR3 | ACCAAGCAGTACCTACGCTAT | TTCTCACCACGAAGTGTCACA |
| GATA4 | CGACACCCCAATCTCGATATG | GTTGCACAGATAGTGACCCGT |
| YAP1 | TAGCCCTGCGTAGCCAGTTA | TCATGCTTAGTCCACTGTCTGT |
| CTGF | ACCGACTGGAAGACACGTTTG | CCAGGTCAGCTTCGCAAGG |
| CYR61 | AAACCCGGATTTGTGAGGT | GCTGCATTTCTTGCCCTTT |

**Table S2. The shRNA sequences.**

| sh-WDR3 #1 | CCGGAGGACAAGCAGAATCACTATTCTCGAGAATAGTGATTCTGCTTGTCCTTTTTTTG |
| --- | --- |
| sh-WDR3 #2 | CCGGCCTGGAATACAAGATACTCTTCTCGAGAAGAGTATCTTGTATTCCAGGTTTTTTG |
| sh-GATA4 #1 | CCGGCCAGAGATTCTGCAACACGAACTCGAGTTCGTGTTGCAGAATCTCTGGTTTTTG |
| sh-GATA4 #2 | CCGGGGACATAATCACTGCGTAATCCTCGAGGATTACGCAGTGATTATGTCCTTTTTG |
| sh-Control | Provided by RIBOBIO. |
| si-Control | Provided by RIBOBIO. |
| si-WDR3 #1 and #2 | Provided by RIBOBIO. |

**Table S3. The primer sequences for ChIP-qPCR.**

| Gene | Forward primer (**5**′ - 3′) | Reverse primer (**5**′ - 3′) |
| --- | --- | --- |
| **YAP1** | CCGTTTACCCCTCTCAAGTG | CTTAAAGCCGCGAGGATAGA |
